# Clinical Features, Surgical Treatment, and Long-Term Outcome of a Multicenter Cohort of Pediatric Moyamoya

**DOI:** 10.3389/fneur.2019.00014

**Published:** 2019-01-22

**Authors:** Jun Zheng, Le-Bao Yu, Ke-Fang Dai, Yan Zhang, Rong Wang, Dong Zhang

**Affiliations:** ^1^Department of Neurosurgery, Beijing Tiantan Hospital, China National Clinical Research Center for Neurological Diseases, Capital Medical University, Beijing, China; ^2^Center of Stroke, Beijing Institute for Brain Disorders, Beijing, China; ^3^Department of Neurosurgery, The Second Hospital of Hebei Medical University, Hebei, China; ^4^Department of Neurosurgery, Xingtai Third Hospital, Shandong, China

**Keywords:** moyamoya disease, pediatric, revascularization, stroke, prognostic factor

## Abstract

**Objective:** This study aims to investigate the clinical features, long-term outcomes, and prognostic predictors of a multicenter cohort of children with moyamoya disease.

**Methods:** A series of 303 consecutive pediatric moyamoya disease (MMD) patients were screened in the present study. The clinical characteristics were retrospectively collected, and long-term outcomes was evaluated. Furthermore, logistic regression analyses were performed to determine the prognostic predictors for the clinical outcome.

**Results:** The mean onset age at diagnosis was 9.4 years old. The gender ratio (girl-to-boy ratio) was 1.1:1.0. Among these 303 patients, 13 patients underwent different surgical modalities in bilateral hemispheres, while eight patients failed to follow-up, and were excluded. Therefore, a total of 282 patients were analyzed. Among these patients, 17 patients underwent combined bypass (CB), 47 patients underwent direct bypass (DB), 150 patients underwent indirect bypass (IB), and 68 patients underwent conservative treatment. Furthermore, recurrent stroke events were observed in 35 patients (12.4%). The Kaplan-Meier analysis demonstrated that there was no significant difference in either ischemia or hemorrhage-free time among the different surgical modalities (*P* = 0.67 and 0.79, respectively). Furthermore, longer ischemia-free time was observed in the surgical group, when compared to the conservative group (*P* < 0.01). In addition, 82.7% (177/214) of patients who underwent surgical treatment obtained good outcomes (mRS 0-1), which were significantly higher than the rate of patients who underwent conservative treatment (52.9%, 36/68; *P* < 0.01). The rate of patients with improved symptoms was also significantly different (93.0 vs. 16.2%, *P* < 0.01). However, no significant difference was observed in the rate of good outcomes, disability, and improved symptoms among the different surgical modalities. The logistic regression analyses revealed that postoperative ischemic events were the only risk factor associated with unfavorable clinical outcome (OR:3.463; 95% CI:1.436–8.351; *P* < 0.01).

**Conclusion:** CB, DB, and IB might have similar effects on long-term clinical outcome in pediatric MMD. However, surgical revascularization is superior, when compared to conservative treatment. Furthermore, postoperative ischemic events were confirmed as potential prognostic factors associated with unfavorable clinical outcome.

## Introduction

Moyamoya disease (MMD) is a progressive narrowing or occlusion cerebrovascular disease characterized by bilateral stenosis of the internal carotid arteries (ICA) and the development of a compensatory network of basal collaterals (1–3). MMD is one of the most common pediatric cerebrovascular diseases in Eastern Asian countries that causes stroke in children (4). The disease manifests in several ways, which involve transient ischemic attack (TIA), cerebral infarction, intracranial hemorrhage, and other types of symptoms (5). Surgical revascularization has been widely accepted for the treatment of MMD to prevent ischemic symptoms (6). In children with MMD, indirect bypass (IB) is more commonly used than direct bypass (DB), which is frequently technically not feasible (7). However, the optimal surgical candidates for children with MMD are not well-identified, and the objective evaluation of the efficacy of surgery and outcomes of conservative treatment is also needed (8–11). Therefore, the data of 303 pediatric MMD patients from a multicenter cohort study conducted between September 2012 and May 2017 were presented. The demographics and clinical characteristics were investigated, and the effects of different surgical modalities and conservative treatment were also evaluated. In addition, a risk factor analysis was conducted to evaluate the independent prognostic factors for clinical outcome.

## Methods

### Patient Selection

This multicenter registry, prospective cohort study on the surgical treatment for MMD was conducted between September 2012 and May 2017 (Registration number: ChiCTR-OCH-12002508). A total of five medical centers were involved in the present study. The study protocol was approved by the Institutional Review Board of all participating hospitals. Furthermore, a written informed consent was obtained from all patients. MMD was diagnosed based on the 2012 guidelines (1) for MMD using digital subtraction angiography (DSA) and/or magnetic resonance angiography (MRA). The inclusion criteria were as follows: (1) patients with a definitive diagnosis of MMD by DSA and/or MRA; (2) patients who were ≤ 16 years old. The exclusion criteria were as follows: (1) patients with moyamoya syndrome secondary to the identified etiologies, including vasculitis, dissection, and tumor; (2) patients in the acute phase (within 3 months) of intracranial hemorrhage or cerebral infarction; (3) patients who suffered from severe cognitive or motor impairment after intracranial hemorrhage or cerebral infarction.

### Clinical Data and Surgical Modalities

Clinical data were prospectively collected, including gender, age, hypertension, smoking history, initial symptoms, Suzuki angiographic stage, neurological status, and procedural complications, after admission. According to the guidelines for MMD (1), the initial symptoms were categorized into seven types: cerebral infarction, intracranial hemorrhage, transient ischemic attacks (TIAs), frequent TIAs (≥2 times per month), headache, epilepsy, and asymptomatic disease. Neurological status was evaluated using the modified Rankin scale (mRS) score on admission and at follow-up. The patients were followed up at the clinic at 3–6 months after surgery and by telephone annually, thereafter. The telephone follow-up was performed by the same physician in each center. Recurrent intracranial hemorrhagic, cerebral ischemic events, and change in symptoms (classified as improved or not improved) during the follow-up period were recorded. The neurological status (mRS score) at the last follow-up was evaluated, and classified as either good (mRS score ≤ 2) or poor (mRS score ≥3).

Three different surgical procedures were performed: combined bypass (CB), DB, and IB. The choice of surgical modality was mainly based on angiographic features and the patient's physical condition. Generally, CB and DB are preferred for most patients, while IB is only performed when the donor or recipient artery is too small or fragile to perform an artery anastomosis. Indirect bypass was placing donor materials such as the anterior and the posterior branches of the STA and the frontal and the temporal muscles directly onto the surface of the ischemic brain included encephalo-duro-arterio-synangiosis (EDAS) and encephalo-myo-arterio-synangiosis (EMAS). The detailed surgical procedures and principles of the surgical strategies are described in the previous studies conducted by the investigators (12, 13). In addition, pediatric patients with conservative treatment were also enrolled in the present study, and these patients were only considered when patients rejected surgical treatment. The main reason why these patients refused the treatment involved economic factors and extreme reluctance to take a risk for operation because most of them presented with mild symptom like TIA. Aspirin was not suggested for those patients with conservative treatment because there was no consensus on the usage of aspirin in pediatric moyamoya while adequate drinking volume was stongerly recommended instead.

### Statistical Analysis

All statistical analyses were performed using SPSS (version 23.0, IBM). Categorical variables were presented as counts (with percentages). Pearson chi-square test, Fisher exact test, or Mann-Whitney *U*-test were used to compare categorical variables, as appropriate. Univariate/multivariate logistic regression was used to explore the relationship between clinical variables and the outcome. The stroke-free time from the recurrent ischemic and hemorrhagic events was estimated using Kaplan-Meier curves with log-rank statistics. A *P*-value < 0.05 was considered statistically significant.

## Results

### Demographics and Clinical Presentation

Between September 2012 and May 2017, a total of 303 consecutive pediatric patients with MMD were enrolled in the present study. The age of these patients at diagnosis ranged within 2–16 years old (mean: 9.4 years old). Patients who were older than 6 years old were the most common (76.9%). The gender ratio (girl-to-boy ratio) of patients was 1.1:1.0. The incidence of familial occurrence was 6.9% (21/303). TIA was the most common clinical manifestation (48.8%), followed by infarction (20.5%), hemorrhage (12.5%), headache (11.9%), seizure (5.3%), and asymptomatic (1%).

According to the DSA and MRA examination findings, 21 patients were diagnosed with unilateral MMD (6.9%), while 282 patients (93.1%) had bilateral involvement. The posterior cerebral artery (PCA) involvement was revealed in 97 patients (32%). Patients with Suzuki angiographic stage III was the most common (38.9%), followed by Suzuki stage II (21.5%) and Suzuki stage IV (20.1%, Table 1).

**Table 1:** Clinical features of 303 children with moyamoya disease at presentation.

**Characteristic**	**Number of cases (%)**
**SEX (*****n*** **=** **303 PATIENTS)**	
Male	144 (47.5)
Female	159 (52.5)
**AGE, YEAR (*****n*** **=** **303 PATIENTS)**	
0–3	12 (4.0)
4–6	58 (19.1)
7–16	233 (76.9)
Familial occurrence (*n* = 303 patients)	21 (6.9)
**INITIAL SYMPTOMS (*****n*** **=** **303 PATIENTS)**	
Transient ischemic attacks	148 (48.8)
Infarction	62 (20.5)
Hemorrhage	38 (12.5)
Headache	36 (11.9)
Seizure	16 (5.3)
Asymptomatic	3 (1.0)
Bilateral lesions (*n* = 303 patients)	282 (93.1)
PCA involvement (*n* = 303 patients)	97 (32.0)
**SUZUKI ANGIOGRAPHIC STAGE (*****n*** **=** **606 HEMISPHERES)**	
I	30 (5.0)
II	130 (21.5)
III	236 (38.9)
IV	122 (20.1)
V	57 (9.4)
VI	10 (1.7)
Normal	21 (3.5)

*PCA, posterior cerebral artery*.

### Surgical Treatment and Procedure-Related Complication

Among the 303 patients, 13 patients underwent different surgical modalities in each hemisphere, while eight patients were lost to follow-up, and were excluded. Therefore, a total of 282 patients were analyzed. Among these patients, 17 patients underwent CB, 47 patients underwent DB, 150 patients underwent IB, and 68 patients underwent conservative treatment. The baseline characteristic was compared between different treatments (Table 2 and Supplemental Table 1). The difference in PCA involvement, mRS score at admission and Suzuki stage of patients between the two groups was not statistically significant. Furthermore, complications were observed in 4.4% of 315 operations, including hyperperfusion in four (1.3%) patients, epilepsy in four (1.3%) patients, a new infarction in two (0.6%) patients, wound infection in two (0.6%) patients, and subdural effusion in two (0.6%) patients.

**Table 2:** Comparison of clinical and imaging features between MMD patients with different management methods.

**Variables**	**CB** **(*n* = 17)**	**DB** **(*n* = 47)**	**IB** **(*n* = 150)**	**Conservative** **(*n* = 68)**	***P* value**
Sex					0.437
Male	11 (64.7)	21 (44.7)	75 (50.0)	30 (44.1)	
Female	6 (35.3)	26 (55.3)	75 (50.0)	38 (55.9)	
Age, year					0.003
0–3	1 (5.9)	1 (2.1)	8 (5.3)	1 (1.5)	
4–6	3 (17.6)	1 (2.1)	28 (18.7)	20 (29.4)	
7–16	13 (76.5)	45 (95.8)	114 (76.0)	47 (69.1)	
Operated hemispheres					0.007
Unilateral	3 (17.6)	29 (61.7)	81 (54.0)	0 (0.0)	
Bilateral	14 (82.4)	18 (38.3)	69 (46.0)	0 (0.0)	
**DISEASE TYPE**					
Infarction	3 (17.6)	9 (19.1)	28 (18.7)	13 (19.1)	0.002
Hemorrhage	0 (0.0)	6 (12.8)	10 (6.7)	19 (27.9)	
Other	14 (82.4)	32 (68.1)	112 (74.6)	36 (52.9)	
PCA involvement	5 (29.4)	12 (25.5)	51 (34.0)	22 (32.4)	0.744
mRS score					0.047
0–1	9 (52.9)	36 (76.6)	116 (77.3)	42 (61.8)	
2	7 (41.2)	11 (23.4)	30 (20.0)	21 (30.9)	
3–5	1 (5.9)	0 (0.0)	4 (2.7)	5 (7.4)	
Suzuki stage					0.068
I	0 (0.0)	0 (0.0)	0 (0.0)	3 (4.4)	
II	1 (5.9)	2 (4.3)	7 (4.7)	8 (11.8)	
III	8 (47.1)	23 (48.9)	64 (42.7)	22 (32.4)	
IV	6(35.3)	16 (34.0)	45 (30.0)	21 (30.9)	
V	2 (11.8)	6 (12.8)	30 (60.0)	13 (19.1)	
VI	0 (0.0)	0 (0.0)	4 (2.7)	1 (1.5)	

*CB, combine bypass; DB, direct bypass; IB, indirect bypass; PCA, posterior cerebral artery; CB involved STA-MCA anastomosis and EDAS or EDMS; DB involved STA-MCA anastomosis; IB involved EDAS or EDMS or multiple bur holes*.

### Follow-Up Outcomes

**Figure 1:**
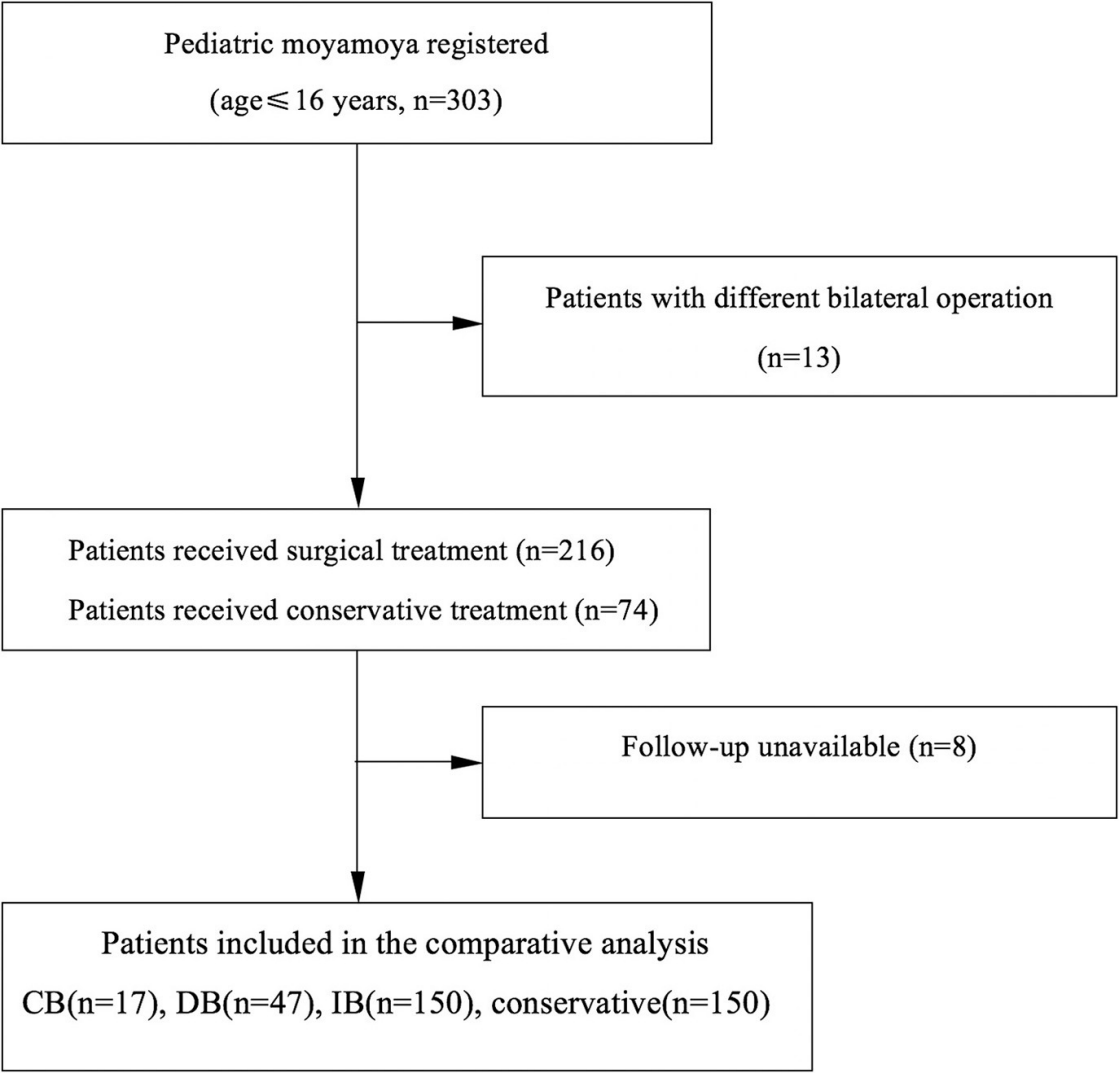
Flow diagram of the study participants.

**Figure 2:**
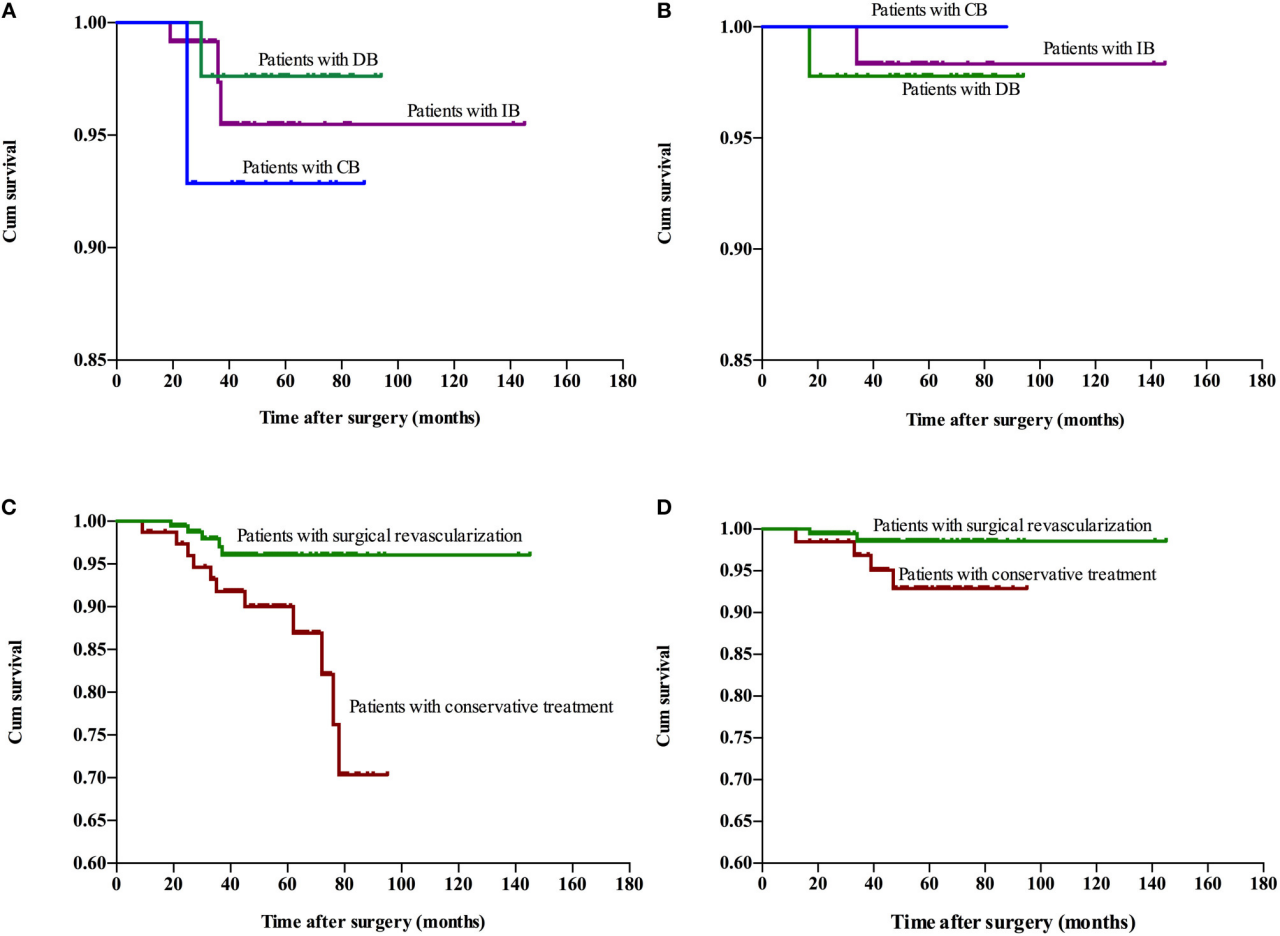
Kaplan-Meier plot for stroke-free survival after surgery. Ischemia-free time **(A)** and hemorrhage-free time **(B)** for patients who underwent different surgical modalities. Ischemia-free time **(C)** and Hemorrhage-free time **(D)** for patients who underwent surgical treatment and conservative treatment. Cum, cumulative.

**Figure 3:**
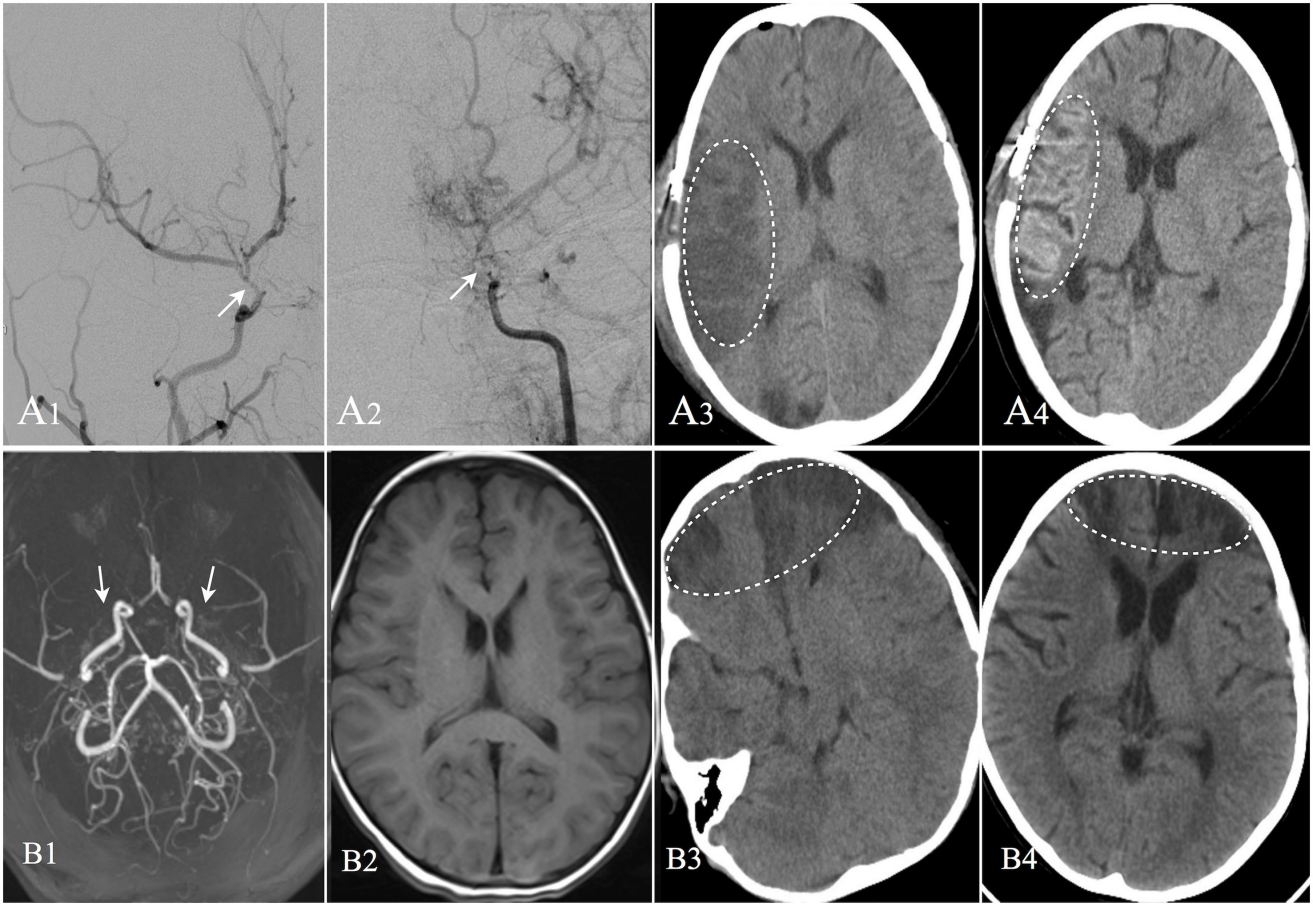
Two cases suffered postoperative operate-related infarction were showed. Patient A: 8 years old male, DSA confirmed moyamoya disease **(A1,A2)**. CT image showed cerebral infarction occurred in the right operative region at the second day after EDAS operation **(A3)**. CT performed at the 13th day after operation showed the infarction region with cortical laminar necrosis **(A4)**. The patient was discharged with weakness in his left limbs; Patient B: 4 years old female, MRA confirmed moyamoya disease **(B1)**. MRI image showed no remote cerebral infarction **(B2)**. CT image showed cerebral infarction occurred in bilateral frontal lobes at the second day after EDAS operation **(B3)**. CT performed at the 20th day after operation showed improvement in the infarction region **(B4)**, and the patient was discharged with weakness in her upper right limb.

The follow-up period ranged within 9–145 months, with a mean period of 41 months involving: CB, ranged from 10 to 88 months with mean 48.4 months; DB, ranged from 10 to 94 months with mean 60.7 months; IB, ranged from 9 to 145 months with mean 34.5 months; Conservative group, ranged from 9 to 95 months with mean 53.3 months. Recurrent stroke events were observed in 35 patients, which involved six patients with hemorrhage and 29 patients with ischemia (TIA in 20 patients and infarction in nine patients). One patients died due to hemorrhage after 27 months of conservative treatment. No significant difference in recurrent stroke events was observed among patients with different surgical modalities, while a higher rate of recurrent stroke events with significant difference was observed in the conservative treatment group, when compared to the surgical treatment group (Table 3). The Kaplan-Meier analysis of recurrent stroke events was performed (Figure 1). The Kaplan-Meier analysis demonstrated that there was no significant difference in either ischemia-free time or hemorrhage-free time among the different surgical modalities (*P* = 0.67 and 0.79, respectively), and there was no significant difference in hemorrhage-free time between the surgical and conservative groups. However, a significant difference was observed in ischemia-free time between the surgical and conservative groups (*P* < 0.01, Figure 2). In addition, 82.7% (177/214) of patients who underwent surgical treatment obtained good outcomes (mRS 0-1), which was significantly higher than the rate of patients who underwent conservative treatment (52.9%, 36/68; *P* < 0.01). Furthermore, the rate of patients with improved symptoms was also significantly different (93.0% vs. 16.2%, *P* < 0.01). However, no significant difference was observed in the rate of good outcomes, disability, and improved symptoms among patients with different surgical modalities (Table 3, Figure 3).

**Table 3:** Comparison of outcomes between MMD patients with different management methods.

	**CB** **(*n* = 17)**	**DB** **(*n* = 47)**	**IB** **(*n* = 150)**	***T*/*X*^**2**^**	***P* value**	**Total surgical revascularization** **(*n* = 214)**	**Conservative treatment** **(*n* = 68)**	***T*/*X*^**2**^**	***P* value**
**FOLLOW-UP EVENTS**									
TIA	1 (5.9)	2 (0.4)	4 (0.3)	1.492	0.517	7 (3.3)	13 (19.1)	19.666	< 0.001
Ischemic stroke	1 (5.9)	1 (0.2)	2 (0.1)	2.99	0.204	4 (1.9)	5 (7.4)	5.023	0.025
Hemorrhage stroke	0 (0.0)	1 (0.2)	1 (0.06)	1.707	0.510	2 (0.9)	4 (5.9)	6.066	0.032
Death due to stroke	0 (0.0)	0 (0.0)	0 (0.0)			0 (0.0)	1 (1.5)	3.158	0.241
**GOOD OUTCOMES**									
mRS 0–1	13 (76.5)	37 (78.7)	127 (84.7)	1.387	0.500	177 (82.7)	36 (52.9)	24.744	< 0.001
mRS 2	3 (17.6)	7 (14.9)	21 (14.0)	0.172	0.918	31 (14.5)	18 (26.5)	5.163	0.023
Disability	1 (5.9)	3 (6.4)	2 (0.1)	4.597	0.092	6 (2.8)	14 (20.6)	24.770	< 0.001
Improved symptoms	14 (82.4)	43 (91.5)	142 (94.7)	3.825	0.113	199 (93.0)	11 (16.2)	160.141	< 0.001

*CB, combine bypass; DB, direct bypass; IB, indirect bypass; PCA, posterior cerebral artery; CB involved STA-MCA anastomosis and EDAS or EDMS; DB involved STA-MCA anastomosis; IB involved EDAS or EDMS or multiple bur holes*.

### Predictors of Surgical Outcome

The univariate logistic regression analysis of the preoperative clinical variables revealed that only postoperative ischemic events [odds ratio (OR): 3.258; 95% confidence interval (CI): 1.366–7.772; *P* < 0.01] were associated with an unfavorable clinical outcome. The other clinical variables, which involved age at onset, gender, initial symptoms, PCA involvement, and Suzuki angiographic stage, were not associated with an unfavorable clinical outcome (Table 4). Next, variables with *P* < 0.2 in the univariate analysis were applied into the multivariate analysis. The multivariate logistic regression analysis revealed that postoperative ischemic events were the only factor associated with unfavorable clinical outcome (OR: 3.463; 95% CI: 1.436–8.351, *P* < 0.01; Table 5).

**Table 4:** Univariate logistic regression analysis for predictive factors of unfavorable surgical outcome.

**Characteristic**	**OR**	**95% CI**	***P* value**
Age at onset	1.065	0.966–1.174	0.209
**SEX**			
Male	1		0.458
Female	1.302	0.648–2.614	
**INITIAL SYMPTOMS**			
**Transient ischemic attacks**			
Absent	1		0.213
Present	1.121	0.357–1.231	
**Infarction**			
Absent	1		0.532
Present	0.879	0.573–1.375	
**Hemorrhage**			
Absent	1		0.871
Present	0.776	1.072–3.472	
**Headache**			
Absent	1		0.432
Present	1.032	0.793–2.359	
**Seizure**			
Absent	1		0.544
Present	1.375	0.491–3.849	
**PCA involvement**			
Absent	1		0.689
Present	1.160	0.561–2.398	
**Suzuki angiographic stage**			
≤ 3	1		0.788
≥4	0.909	0.455–1.816	
**Postoperative ischemic events**			
No	1		0.008
Yes	3.258	1.366–7.772	

*OR, odds ratio; CI, confidence interval; PCA, posterior cerebral artery*.

**Table 5:** Multivariate logistic regression analysis for predictive factors of unfavorable surgical outcome.

**Characteristic**	**OR**	**95% CI**	***P* value**
Age at onset	1.077	0.973–1.192	0.150
**POSTOPERATIVE ISCHEMIC EVENTS**			
No	1		0.006
Yes	3.463	1.436–8.351	

*OR, odds ratio; CI, confidence interval*.

## Discussion

Moyamoya Disease (MMD) is mainly found in Asia, especially in Japan. Meanwhile, in recent decades, more and more patients with MMD have been found in China (14, 15). The data obtained from a recent multicenter cohort of 303 cases of pediatric MMD was presented. The present study is among the largest series that focused on the clinical feature, clinical outcomes, and predictive variables of the clinical outcome of children with MMD (14, 16, 17). A comparative analysis of the effects of different surgical modalities on clinical outcome was also performed. The present study demonstrated that TIA was the most common clinical manifestation, and that bilateral MMD occurred in most pediatric patients. Pediatric patients with Suzuki angiographic stage III was the most common, followed by Suzuki stage II. In addition, there were no significant differences in recurrent stroke events, ischemia-free time, or hemorrhage-free time, and in the proportion of patients with good outcome or improved symptoms, who underwent different surgical modalities. However, surgical revascularization could significantly reduce recurrent stroke events, extend ischemia-free time, improve patient symptoms, and obtain a good outcome, when compared with conservative treatment. Furthermore, postoperative ischemic events were found to be the only factor associated with unfavorable clinical outcome.

Surgical revascularization was demonstrated as the only effective treatment for MMD, when compared to medical treatment or conservative treatment, since there is no definitive medical treatment to stabilize the course of MMD (18–20). The present results confirm these previous reports, in which surgical revascularization could significantly reduce recurrent stroke events and improve patient symptom, when compared with conservative treatment, in pediatric MMD. However, the optimal surgical procedure for pediatric MMD has been less documented. Bao et al. (14) reported a large single-center cohort of pediatric MMD patients. However, merely indirect revascularization was performed, since they considered that direct revascularization was frequently technically not feasible, while the response to indirect revascularization was excellent. Furthermore, their long-term survey demonstrated that most surgically treated pediatric patients with MMD maintained good outcomes (86%). In a previous study conducted by the investigators (12), in which a single-center cohort compared long-term outcomes among different surgical modalities in pediatric patients with MMD, it was demonstrated that there were no significant differences among the different surgical modalities in terms of the incidence of good neurological status (*P* > 0.999) or symptom improvement rate (*P* = 0.58). However, the previous study was a relatively small cohort, which comprised of 114 pediatric MMD patients. Therefore, a large multi-center cohort analysis was presented in the present study. These present results revealed that there was no significant difference in long-term outcomes among the different surgical modalities, which strongly suggests that indirect revascularization is good enough to improve cerebral hemodynamics and reduce the incidence of subsequent ischemic events in pediatric MMD.

In previous reports (11, 12, 14, 18, 20), nearly 8–20% of pediatric MMD patients failed to obtain a favorable clinical outcome following a revascularization operation. The prognostic factors for pediatric MMD patients, which involved preoperative multiple cerebral infarctions, early onset at a young age, high Suzuki stages on cerebral angiography, and perioperative ischemic events, were identified in previous studies. Kim et al. (9) found that infarction on presentation was associated with unfavorable clinical outcome (OR: 2.85; 95% CI: 1.49–5.46; *P* < 0.01) and decreased vascular reserve only on single-photon emission computerized tomography (OR: 0.07; 95% CI: 0.01–0.52; *P* < 0.01), which has a favorable clinical outcome. Bao et al. (14) reported that younger age of symptom onset and postoperative ischemic events were identified as predictors of unfavorable clinical outcome, and this was consistent with the present results, showing that postoperative ischemic event was the only prognostic factor associated with unfavorable clinical outcome. Therefore, careful attention should be given to avoid postoperative ischemic events, since these were the most common perioperative complications closely correlated to unfavorable clinical outcome. Postoperative ischemic events might be associated with many factors, which involve the surgical procedure itself, the anesthesia process, and hemodynamic status during the perioperative period, and these needs further research (21–24).

There were several limitations in the present study. First, this was not a randomized controlled trial, since a randomized trial was difficult to perform. Furthermore, selection bias for the different surgical procedures existed. In addition, the baseline features were not completely comparable. Thus, potential confounding factors might have influenced both the surgical choices and outcomes. Moreover, follow-up duration of conservative group was relatively less than interventions groups which might underestimate the severity of the outcome in the conservative as compared to the intervention group. Second, the subgroup analysis for hemorrhage and infarction was not performed due to the limited volume of cases with hemorrhage. Third, predictive factors for postoperative ischemic events were not analyzed in the present study, which needs further research. Fourth, the present study was devoted to MMD. In non-Asian children, the rate of MMS/MMD is indeed higher, and perhaps the present results are not completely applicable to children with an underlying etiology.

## Conclusion

The present prospective multi-center cohort study demonstrated that CB, DB, and IB might have similar effects on long-term clinical outcome in pediatric MMD. However, surgical revascularization is superior in improving symptoms and preventing recurrent ischemic stroke events, when compared to conservative treatment. Postoperative ischemic events were confirmed as the potential prognostic factor associated with unfavorable clinical outcome.

## Ethics Statement

This study was carried out in accordance with the recommendations of the ethics committee of Beijing Tiantan Hospital with written informed consent from all subjects. All subjects gave written informed consent in accordance with the Declaration of Helsinki. The protocol was approved by the the ethics committee of Beijing Tiantan Hospital.

## Author Contributions

JZ, L-BY, K-FD, YZ, RW, and DZ have substantially contributed to the conception, design, analysis, and interpretation of the data as well as to drafting the article and revising it critically. All authors have read and approved the final version of the manuscript.

### Conflict of Interest Statement

The authors declare that the research was conducted in the absence of any commercial or financial relationships that could be construed as a potential conflict of interest.
