# Supplementary material for: Clinical Features, Surgical Treatment, and Long-Term Outcome of a Multicenter Cohort of Pediatric Moyamoya
Source: Front Neurol. 2019 Jan 22;10:14. doi: 10.3389/fneur.2019.00014 (PMC6349739; doi:10.3389/fneur.2019.00014)
Supplement: Supplementary file 1 [file Table_1.DOC]

Supplemental Table 1. Comparison of clinical and imaging features between MMD patients from different participating centers.

| Variables | Center 1  (n=172) | Center 2  (n=23) | Center 3  (n=43) | Center 4  (n=28) | Center 5  (n=16) | P value |
| --- | --- | --- | --- | --- | --- | --- |
| Sex |  |  |  |  |  | 0.754 |
| Male | 81 (47.1) | 13 (56.5) | 19 (44.2) | 16 (57.1) | 8 (50.0) |  |
| Female | 91 (52.9) | 10 (43.5) | 24 (55.5) | 12 (42.9) | 8 (50.0) |  |
| Age, year |  |  |  |  |  | 0.516 |
| 0-3 | 6 (3.5) | 2 (8.7) | 1 (2.3) | 2 (7.1) | 0 (0.0) |  |
| 4-6 | 29 (16.9) | 5 (21.7) | 11 (25.6) | 3 (10.7) | 4 (25.0) |  |
| 7-16 | 137 (79.7) | 16 (69.6) | 31 (72.1) | 23 (82.1) | 12 (75.0) |  |
| Operated hemispheres | |  |  |  |  | <0.001 |
| Unilateral | 86 (50.0) | 3 (13.0) | 9 (20.9) | 6 (21.4) | 9 (56.3) |  |
| Bilateral | 58 (33.7) | 10 (43.5) | 18 (41.9) | 11 (39.3) | 4 (25.0) |  |
| Disease type |  |  |  |  |  |  |
| Infarction | 20 (11.6) | 5(21.7) | 14 (32.6) | 8 (28.6) | 6 (37.5) | <0.001 |
| Hemorrhage | 13 (7.6) | 2 (8.7) | 10 (23.3) | 8 (28.6) | 2 (12.5) |  |
| Other | 139 (80.8) | 16 (69.6) | 19 (44.2) | 12 (42.9) | 8 (50.0) |  |
| PCA involvement | 51 (29.7) | 9 (39.1) | 18 (41.9) | 7 (25.0) | 5 (31.3) | 0.744 |
| mRS score |  |  |  |  |  | 0.502 |
| 0-1 | 131 (76.2) | 15 (65.2) | 26 (60.5) | 19 (67.9) | 12 (75.0) |  |
| 2 | 36 (20.9) | 7 (30.4) | 14 (32.6) | 8 (28.6) | 4 (25.0) |  |
| 3-5 | 5 (2.9) | 1 (4.3) | 3 (7.0) | 1 (3.6) | 0 (0.0) |  |
| Treatments |  |  |  |  |  | 0.016 |
| CB | 9 (5.2) | 3 (13.0) | 1 (2.3) | 3 (10.7) | 1 (6.3) |  |
| DB | 33 (19.2) | 2 (8.7) | 7 (16.3) | 3 (10.7) | 2 (12.5) |  |
| IB | 102 (59.3) | 8 (34.8) | 19 (44.2) | 11 (39.3) | 10 (62.5) |  |
| Conservative | 28 (16.3) | 10 (43.5) | 16 (37.2) | 11 (39.3) | 3 (18.8) |  |

CB=combine bypass; DB=direct bypass; IB=indirect bypass; PCA=posterior cerebral artery; CB involved STA-MCA anastomosis & EDAS or EDMS; DB involved STA-MCA anastomosis; IB involved EDAS or EDMS or multiple bur holes.
